# Appointment

**Published:** 1871-09

**Authors:** 


### Appointment.

Dr. Worthington W. Miner, of Ware, Mass., of the class of 1868, at Amherst, and a graduate of the Buffalo Medical College of 1871, has been appointed Assistant Physician at the Butler Insane Asylum, Providence, R. I., and entered upon his duties in August last. Many of our readers will remember his reports of cases, last season published in this Journal, and his many friends in Buffalo and elsewhere, will be pleased to learn of his success.

